# Addressing Occupational Back Pain: A Systematic Review of Preventive and Therapeutic Strategies

**DOI:** 10.7759/cureus.48744

**Published:** 2023-11-13

**Authors:** Dania Gari, Abdulhadi A Alabdulhadi, Abdulrahman A Alahmari, Zahrah A Alsalman, Hani S Alshehri

**Affiliations:** 1 Family and Community Medicine, Imam Abdulrahman Bin Faisal University, Khobar, SAU; 2 Home Health Care, Prince Saud Bin Jalawi Hospital, Alahsa, SAU; 3 Family Medicine, Eastern Health Cluster, Khobar, SAU; 4 Bariatric Surgery Center, Prince Saud Bin Jalawi Hospital, Alahsa, SAU; 5 Family and Community Medicine, Khobar Health Network, Eastern Health Cluster, Ministry of Health, Khobar, SAU

**Keywords:** occupational back, therapeutic options, prevention, cervicobrachial disorders, work-related lower back pain

## Abstract

Occupational back pain has emerged as a significant public health concern. Despite several efforts to mitigate the adverse effects of occupational back pain, this issue still persists across the globe. This systematic review summarizes the preventive and therapeutic options available for managing occupational back pain. A systemic search was carried out in various databases including PubMed, Web of Science, CINAHL Ultimate, and Scopus to identify relevant literature. The search was also extended to Google Scholar to identify more relevant studies. A combination of keywords was used during the search. Studies were included if they focused on occupational back pain, investigated preventive and treatment options, and were published in English. A total of 20 relevant studies, including 62,176 participants, were included in this systemic review. Out of these 20 studies, 10 were randomized control trials, four were cross-sectional studies, two were longitudinal studies, one was a single-blinded clinical study, two were prospective studies, and the remaining one was a pilot study. This systemic review identified various interventions to improve occupational back pain. The common therapeutic strategies included educational programs, physio and rehab interventions, acupuncture, mixed treatment strategies, reflexology, massage, yoga, active physical therapy, and real-time occupational internet-based interventions. Some studies also reported the effectiveness of opioid therapy and non-steroidal anti-inflammatory drugs for managing back pain. Findings indicated that these therapies effectively reduced occupational back pain and improved quality of life. However, opioid therapy uses also raised safety concerns. The findings of this systemic review highlight the importance of adopting evidence-based strategies to address occupational back pain effectively.

## Introduction and background

Occupational back pain has emerged as a significant issue that affects millions of workers worldwide [1]. According to the World Health Organization (WHO), back pain is the major contributor to disability in the world and most people are likely to experience back pain during their lives. The highest incidence of back pain is reported between 50 and 55 years [2]. Despite several efforts to mitigate the impact of back, this phenomenon still has a considerable impact on individuals, employers, and society. An estimate suggests that almost one in four individuals are affected by work-related back pain [3]. Similarly, surveys involving material handling workers reported that a 12-month incidence of low back pain, which lasted for seven days was 25% of respondents. Furthermore, 14% of these individuals sought medical care [4]. Occupational back pain afflicts individuals from all fields of life regardless of their professions, industries, and age groups. The human spine is a marvel of engineering that is designed to provide flexibility and support to the body. However, the modern workplace has seen a shift towards sedentary lifestyles, repetitive tasks, prolonged sitting, and improper ergonomics that can lead to occupational back pain [5]. Although some back pain can be attributed to work-related demands; however, non-work-related back pain can also contribute towards the overall burden of this condition. Therefore, it usually becomes difficult to ascertain the exact cause of back pain. 

In most cases, back pain can resolve on its own; however, this experience can still have an adverse impact on occupational performance. The consequences of occupational back pain extend beyond mere physical discomfort. Workers experiencing back pain have reduced mobility, decreased flexibility, and compromised quality of life [6]. Furthermore, such individuals can have sleep disturbances and psychological distress. A survey by Serranheira et al. that included 735 workers reported that a 12-month rate of absenteeism was 4% in respondents. Furthermore, they found that the risk of absenteeism was higher in individuals who had more physically demanding work compared to those who had a sedentary work environment [7]. This affects not only individuals but also the performance of the organization they work in. This ultimately has severe financial implications for the countries. According to an estimate, almost 80 billion dollars are spent in the United States on lower back pain, which accounts for roughly 156 million lost working days [8]. The management of occupational back pain is a multifaceted approach that involves both workers and employers. Adopting preventive interventions can help reduce the higher incidence of work-related back pain [9]. Understanding the root cause of occupational back pain and its preventive measures can pave the way for healthier and more productive workplaces. This systemic review summarizes the current knowledge regarding occupational back pain and various treatment and management options available.

## Review

Methodology

This systemic review was conducted per the Preferred Reporting Items for Systematic Reviews and Meta-Analyses (PRISMA) guidelines [10].

Search Strategy and Data Sources

To identify the relevant studies relating the occupational back pain, a systemic search was carried out in various databases. A separate search was undertaken in PubMed, Web of Science, CINAHL Ultimate, and Scopus databases to find relevant studies. A combination of keywords, such as "work-related low back pain," "occupational cervicobrachial disorders," and "work-related musculoskeletal disorders," along with terms like "prevention" or "therapeutic options," was used (Appendix). Furthermore, alternatives to these keywords were also used in the search strategy. To further increase the number of potential studies, Google Scholar was also searched. The studies were considered for inclusion if they met the following criteria: (1) they studied occupational back pain, (2) they investigated the preventive and treatment options for occupational back pain, and (3) the publications were in English language.

In this study, the researchers used specific tools to assess the quality of different types of studies based on their designs. To evaluate randomized controlled trials (RCTs), the researchers employed the Cochrane Risk of Bias Tool, a commonly used tool for assessing methodological rigor and potential biases in RCTs. For observational studies, on the other hand, the researchers utilized the Newcastle-Ottawa Scale, another widely used tool that helps evaluate the quality of such studies. By employing these tools, the researchers were able to comprehensively and rigorously evaluate the methodological quality and potential biases of the different study designs, ensuring a thorough assessment of the evidence.

Data Collection Process

The search results from each database were sent to EndNote (a reference manager; Clarivate Analytics, Philadelphia, PA). At this stage, duplicates were removed before being exported to Rayyan (systemic review screening software; Rayyan Systems Inc., Cambridge, MA) [11]. Two reviewers were tasked with data extraction. The *blind* was turned on for both reviewers to ensure that the results had no bias. In the first step, both reviewers carried out study selection based on the title and abstract of the studies. After the exclusion of nonrelevant articles, the reviewers performed a thorough analysis of each article to ensure that predetermined inclusion criteria were met. In cases of disagreement between reviewers, a third reviewer was involved to resolve the issue. Finally, all the relevant data, including demographics, outcomes, and results of each study, were obtained.

Flow Diagram 

**Figure 1:**
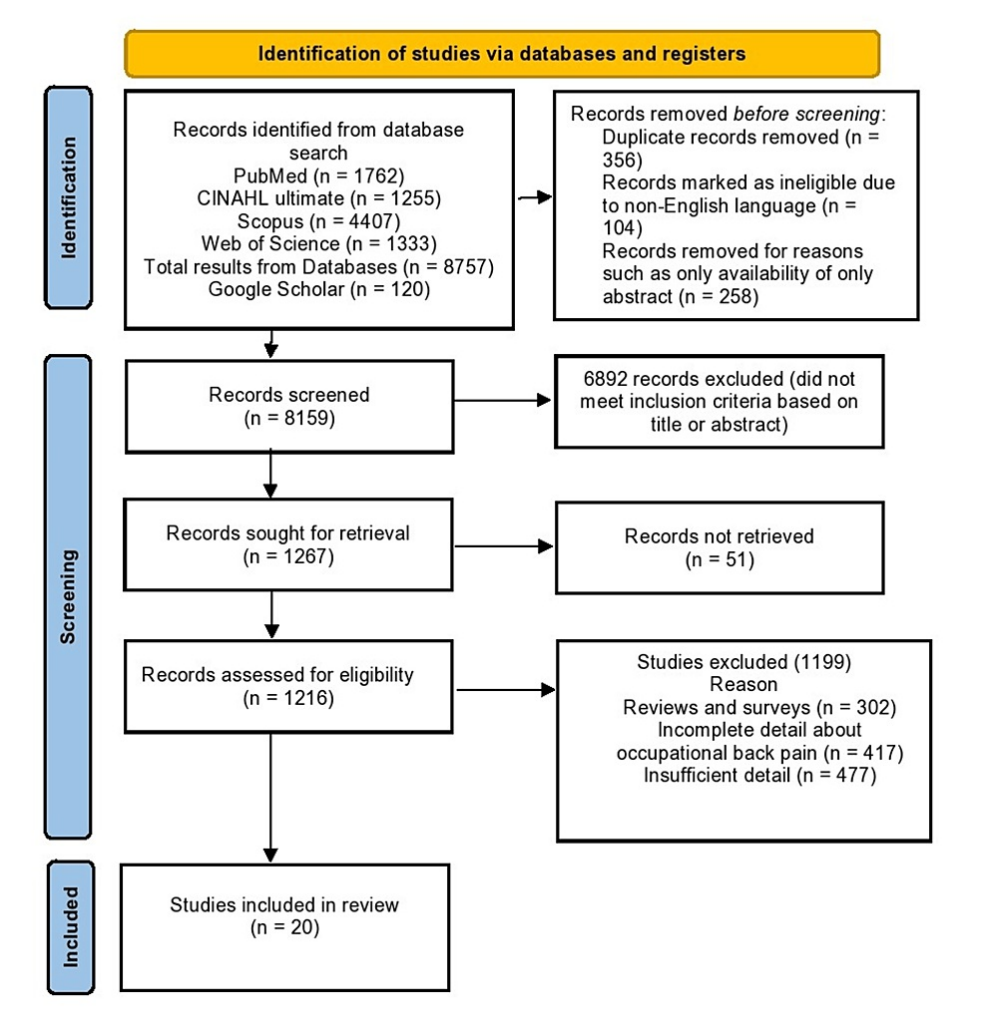
PRISMA flow diagram of the systemic review. PRISMA, Preferred Reporting Items for Systematic Reviews and Meta-Analyses

Figure 1 shows the PRISMA flow diagram of the systemic review. The figure explains the reasons for the exclusion of nonrelevant studies.

Results

Included Studies

A total of 8,877 potential studies were identified during the database search. The number of studies identified from each database was as follows: PubMed (*n* = 1,762), CINAHL Ultimate (*n* = 1,255), Web of Science (*n* = 1,333), and Scopus (*n* = 4,407). The research in Web of Science was refined by limiting the search to back pain. The research in Scopus was limited to five years only. The search in Google Scholar revealed 120 studies. Following the removal of redundant studies and publications not written in the English language, a total of 8,159 records were subjected to further analysis. A total of 6,892 publications were excluded from the scope of this review based on an analysis of keywords and abstracts. A comprehensive evaluation was conducted on the remaining body of literature which led to the selection of 20 studies.

Study Characteristics

The data extracted from all of the studies are given in Table 1. A total of 62,176 participants were included in the 20 studies analyzed in this systematic review. Out of these 20 studies, 10 were RCTs [12-20], four were cross-sectional studies [21-24], two were longitudinal studies [25,26], one was a single-blinded clinical study [27], two were prospective studies [28,29] and the remaining one was a pilot study [30]. Out of these 20 studies, five studies explored the use of educational programs for awareness among patients and caregivers regarding occupational back pain. These studies suggested that education-based programs are effective in raising awareness and preventing people from having occupational back pain. In one of these five studies, it was documented that the Occupational Low Back Pain Prevention Behavior Questionnaire is a reliable way to develop educational interventions among clinical nurses [21].

A study was conducted by Patel et al. to investigate the efficacy of using muscle relaxants and non-steroidal anti-inflammatory drugs (NSAIDs) for the management of low back pain. The results of this study suggested that chlorzoxazone used in combination with ibuprofen is an effective treatment for the management of low back pain [28]. In a study conducted by Tetsunaga et al., the effectiveness of NSAIDs was compared with tramadol-acetaminophen for treating patients with low back pain with depression. It was concluded that tramadol-acetaminophen is more effective than NSAIDs for this purpose [29]. In a randomized trial, the efficacy of acetaminophen for treating low back pain was compared with loxoprofen by Miki et al. The results of this study indicate that acetaminophen is more effective than loxoprofen and is a valuable first-line treatment for acute low back pain management [20].

In their study, Rantonen et al. established that physio and rehab interventions are effective for lowering physical impairment and back pain and improving quality of life [19]. A pilot study by Fox et al. investigated the effectiveness of battlefield acupuncture for lowering back pain in the emergency department. Their findings showed that this approach can significantly reduce low back pain in patients in emergency departments [30]. However, one study showed that multidisciplinary is better in managing low back pain. The authors believed that disparate treatments can impact the attitudes and beliefs of individuals [12]. In a single-blinded randomized clinical trial, it was documented that pressure on H7, GV20, K1, GV20, GB30, BL32, and BL60 can effectively reduce the severity of chronic low back pain in patients [13]. Babadi et al. concluded that reflexology is also an effective management option for chronic back pain [27]. 

Borges et al. reported that massage is effective in lowering occupational back pain [15]. In their study, Saper et al. regarded yoga to be an effective option [16]. Similarly, the use of active physical therapy for improving posture and lowering back pain in clinical nurses was documented in one single-blinded RCT [18]. del Pozo-Cruz et al. investigated the effectiveness of real-time occupational internet-based interventions in preventing chronic low back pain among office workers [17]. It was concluded that internet-based interventions help prevent chronic low back pain in office workers with subacute low back pain. The use of opioid therapy was investigated in two of the studies [26,31]. It was concluded that opioid therapy is an effective treatment option for low back pain, but it has safety concerns as well. In a cross-sectional study, it was documented that low back pain is highly prevalent among teachers, with a higher incidence observed in high school teachers compared to primary school teachers [24].

**Table 1:** The characteristics of included studies in the systemic review. NSAIDs, non-steroidal anti-inflammatory drugs

Study	Year	Study design	Study group	Control group	Main findings	Conclusions
Liu et al. [21]	2023	Multicenter cross-sectional study	1,331	N/A	A total of 86.7% of nurses who experienced low back pain had undergone training specifically targeting low back pain, whereas the remaining 13.3% of nurses did not participate in such training. The preceding group exhibited a greater capacity for mitigating occupational back pain compared to the subsequent group.	Training programs should be conducted for nurses to increase their ability to prevent occupational back pain.
Zhang et al. [22]	2022	Multicenter cross-sectional study	1,186	N/A	The study yielded split-half reliability and test-retest reliability coefficients of 0.663 and 0.734, respectively.	The validity and reliability of the Occupational Low Back Pain Prevention Behavior Questionnaire have been established among clinical nurses.
Alghadir et al. [25]	2021	Cohort study	116	N/A	The perceived knowledge score about occupational low back pain was improved (81.6 ± 18.2) in nurses after six months and was higher than the baseline score (68.2 ± 19.2). The prevalence score of occupational back pain was reduced from 71.5% to 65.0%.	After attending an ergonomic workshop, the knowledge of nurses regarding occupational back pain was improved and the occupational back pain score was reduced.
Iwakiri et al. [23]	2019	Cross-sectional study	2,712	N/A	Within the past year, a significant proportion of care providers, specifically 34.7%, reported experiencing low back pain. A significant correlation was identified between care methods and the occurrence of severe low back pain.	The risk of low back pain among caregivers can be reduced by training and consultation regarding the care methods.
Patel et al. [28]	2019	Open-label, prospective, multicenter study	406	N/A	Chlorzoxazone combined with ibuprofen is effective for low back pain management. The adverse events of this study include headache, fever, gastritis, cold, and stomach pain.	The combination of muscle relaxants with NSAIDs can significantly reduce low back pain.
Rantonen et al. [19]	2018	Randomized controlled trial	126	50	The use of active interventions for low back pain was effective in decreasing physical impairment and increasing the quality of life at two years. During these interventions, no adverse events were observed.	Low back pain in employees can be identified by a health survey and can be managed by active interventions.
Miki et al. [20]	2018	Randomized trial	140	N/A	Acetaminophen is more effective than loxoprofen for treating low back pain. Side effects include drowsiness, gastrointestinal disorders, and leg edema.	Acetaminophen is an effective first-line treatment for the management of acute low back pain.
Fox et al. [30]	2018	Pilot study	15	15	The low back pain numeric pain rating scale was lower in the battlefield acupuncture group as compared to the control group ((5.2 vs. 6.9, *P* = 0.04). The Get-Up-and-Go test was the same for both of the groups (21.3 s vs. 19.0 s, *P* = 0.327).	Battlefield acupuncture is an effective therapy for low back pain in the emergency department.
Ronzi et al. [12]	2017	Monocentric randomized controlled trial	159	N/A	A decrease in sick leave was observed in three groups after 12 months of treatment. No significant difference was observed in the evolution of social ability, personal beliefs, and quality of life among the three groups. No adverse events were observed during the study.	Disparate treatments can show similar effectiveness in patients with low back pain as they work through changes in attitudes, coping mechanisms, and beliefs.
Movahedi et al. [13]	2017	Single-blinded randomized clinical trial	50	N/A	There was no statistically significant difference between the groups in terms of height, body mass index, weight, and age (*P *> 0.05). There was no statistically significant difference observed between the two groups in relation to pain severity before the implementation of the intervention. Following the intervention, the experimental group exhibited a significantly lower average pain score compared to the sham group (*P *= 0.000).	Acupressure is an effective way to reduce the severity of pain in nurses with chronic low back pain.
Babadi et al. [27]	2016	Single-blind clinical trial study	25	25	Before the intervention, no statistically significant difference in pain severity score was present between the two groups (*P *> 0.05). There was a significant difference in pain severity scores between the two groups after intervention (*P *> 0.001).	Reflexology is an effective way to reduce pain intensity scores in patients with chronic low back pain.
Sharafkhani et al. [14]	2016	Randomized controlled trial	50	50	There was not any significant difference between the posttest and pretest knowledge scores of the control group as *P *> 0.05. Low self-efficiency was observed among the members of the control group for adopting preventive measures for chronic low back pain. The health belief subscale scores were higher after intervention than before intervention as *P *< 0.0001.	Educational program based on the Health Benefit Model (HBM) is effective in improving the scores of knowledge among nurses. Educational strategies based on theory are better than traditional educational interventions.
Lee et al. [26]	2016	Retrospective cohort study	2,887	N/A	The total medical cost was higher in the early opioid group. There was an increased risk of long-term opioid use in patients with acute occupational low back pain who received opioids in the emergency department.	Early use of opioids in emergency departments in patients with acute occupational low back pain has adverse outcomes such as increased medical cost and long-term opioid use.
Tetsunaga et al. [29]	2015	Prospective study	95	N/A	Tramadol-acetaminophen is more effective than NSAIDs for treating low back pain and reducing depression. The side effects during this study include weight loss, dizziness, constipation, and nausea.	Tramadol-acetaminophen is an effective drug for the treatment of low back pain in patients with depression.
Borges et al. [15]	2014	Randomized controlled trial	29	14	There was a significant decrease in pain scores on the first and second evaluations in the massage group (*P *< 0.001). Drop intensity was less from the second to the third evaluation (*P *< 0.001). The decrease in pain was subtle from the first to second evaluation in the laser group (*P *> 0.05). Until the third evaluation, there was a discrete continuity in drop (*P *> 0.05). Pain intensity value was increased between the evaluations among the control group (*P *> 0.05).	Massage is an effective way to manage occupational low back pain in nurses.
Bandpei et al. [24]	2014	Cross-sectional study	620	N/A	The rate of response from teachers was 95%. The study found that high school teachers exhibited a statistically significant higher susceptibility to experiencing lower back pain compared to primary school teachers (*P *< 0.05). There was no statistically significant correlation found between the prevalence of lower back pain and exercise (*P *= 0.26). A significant correlation was observed between the prevalence of lower back pain and various factors, including body mass index, length of employment, age, and job satisfaction (*P *< 0.05).	Low back pain is highly prevalent among teachers, with a higher occurrence observed in high school teachers compared to primary school teachers.
Saper et al. [16]	2013	Parallel randomized dosing trial	95	N/A	Back-related function and pain were observed to be improved in both groups (*P *< 0.001). Thirty adverse events were observed in 27% and 34% of individuals in once-weekly and twice-weekly groups, respectively (*P *= 0.47).	In the underserved population with chronic back pain, the effectiveness of once-weekly and twice-weekly yoga classes was similar.
del Pozo-Cruz et al. [17]	2012	Randomized controlled trial	50	50	The statistical difference between the control and intervention groups was nonsignificant at baseline. No adverse events were observed during the trial. For the online occupational exercise intervention group, the compliance rate was 92%.	The progression of subacute nonspecific low back pain to chronicity can be prevented by using real-time occupational internet-based intervention among office workers.
Jaromi et al. [18]	2012	Single-blinded randomized controlled trial	56	55	Back pain was significantly decreased in both groups after therapy. Better results were observed in the back school group during the six-month and one-year follow-up period. A significant improvement was observed in the back school group as compared to the control group by biomechanical analysis of postures after therapy.	Active physical therapy methods can be used to improve posture and lower pain intensity in nurses with chronic low back pain.
Kobus et al. [31]	2012	Prospective study	26,014	N/A	Higher-dose opioid medication users were observed to report poorer health. They also had higher rates of substance use and mental disorders.	Higher dose opioid therapy is being used for 8.6% of the patients with back pain, but it has safety concerns.

Discussion

Occupational back pain is a growing concern and affects several people worldwide. Although several preventive measures are available to reduce the incidence of occupational back pain, the knowledge of this aspect is usually poor in the general public. Therefore, this systemic review was conducted to summarize different treatment options available that can effectively manage occupational back pain. Previously, studies have shown that occupational back pain can be effectively managed by using multidisciplinary approaches [32,33].

In a total of five studies included in our systematic review, it was found that educational interventions and awareness programs help to reduce the number of cases of occupational back pain. Our findings are supported by Lim et al., who recommended the use of educational interventions for the prevention of low back pain [34]. The role of exercise in reducing back pain has been reported previously [35,36]. The beneficial effect of exercise is attributed to its capacity to enhance muscular strength, providing vital support for the lumbar spine. Combining general exercise with various aerobic fitness can serve as an effective approach to the recovery of occupational back pain [37]. In this systemic review, yoga was regarded as an effective therapeutic option for the management of occupational back pain. Similarly, a systemic review and meta-analysis by de Campos et al. reported that educational programs combined with exercise help to improve low back pain intensity and disability associated with it. However, the use of these strategies for the prevention of low back pain and improving quality of life was found to be uncertain [38]. Similarly, Hayden et al. reported in their review that exercise therapy is found to be effective for the management of chronic low back pain [39].

We also found evidence that reported the effectiveness of battlefield acupuncture for treating low back pain. The effectiveness of acupuncture was also documented by Urits et al. They reported that the use of electrically stimulated needles helps to relieve chronic low back pain [40]. Massage is another intervention that can be effective for lower back pain. Our findings are consistent with a meta-analysis and systematic review conducted by Craige et al. They investigated the impact of nonpharmacological interventions such as massage on the sleep of patients with low back pain and found it to be effective [41]. The importance of massage for improving the condition of patients with low back pain was also documented in another study conducted by George et al. [42]. 

Patients require proper guidance and consultation regarding the management of occupational low back pain. The importance of consultation for the management of patients with low back pain is also emphasized in a systematic review conducted by Lim et al. Moreover, the training of healthcare providers is also important in this regard [34]. Our analysis suggests that the use of opioid therapy can be an option for treating low back pain but several risk factors are associated with its use such as long-term opioid use and an increased medical cost. These findings are also supported by a systematic review conducted by Karmali et al. They identified that set criteria should be defined regarding the consistent use of opioid treatment for pain management. Risk factors such as overdose, misuse, and abuse must also be taken into account while using this treatment option [43]. 

Patients’ beliefs and attitudes toward pain perception have a significant effect on pain management. In a study conducted by Main et al., it was concluded that the beliefs of patients are important for the perception of pain and response toward pain. They found that advanced technology can be used to introduce novel intervention approaches to target the expectations and beliefs of patients. It can assist in better psychological adjustments and enhance pain control [44]. We also identified that internet-based interventions can be utilized to prevent chronicity in subacute low back pain in office workers. However, a meta-analysis conducted by Dario et al. suggested that telehealth interventions alone are not very effective for low back pain management [45]. Henrotin et al. investigated the significance of information for the management of low back pain. They demonstrated that multimedia campaigns and internet-based interventions play an important part in raising awareness, management, and prevention of low back pain among individuals [46]. We identified reflexology as an important technique for pain management in patients with low back pain. This evidence is also supported in a review conducted by Stephenson and Dalton. They identified reflexology as an alternative and complementary therapy for pain management. They concluded that it is a nonpharmacological and noninvasive therapy for pain management, but there is a lack of research in this field. Only a few studies have figured out the use of reflexology for pain management. More systematic research is required to investigate the effectiveness of this intervention for pain management [47].

In this study, it is documented that acetaminophen is effective for the treatment of low back pain. Similar results have been supported in a meta-analysis conducted by De Oliveria Jr et al. [48]. These findings have also been propagated in a review conducted by Peck et al. [49]. We have also demonstrated that the combination of NSAIDs and muscle relaxants is effective for the treatment of low back pain. In a meta-analysis conducted by Gianola et al., similar findings have been indicated for the treatment of back pain [50].

## Conclusions

In conclusion, this systemic review offers substantial evidence regarding management approaches for the treatment of occupational back pain. Physiotherapy and rehabilitation interventions were found to effectively reduce physical impairment and back pain. Other interventions such as battlefield acupuncture, reflexology, massage, yoga, and active physical therapy also showed positive outcomes. This review also found that opioid use can be a viable option; however, the adverse effects of these drugs should be monitored. Overall, this systemic review contributes valuable insights into the management of occupational back pain.

## Appendices

**Table 2:** Terms and strategy used for literature search.

#1 Work-related low back pain [TI/AB]	#11 Preventive measures [TI/AB]
#2 Work-related Musculoskeletal Disorders [TI/AB]	#12 Prophylaxis [TI/AB]
#3 Repetitive motion injuries [TI/AB]	#13 treatment [TI/AB]
#4 Repetitive strain injuries [TI/AB]	#14 therapies [TI/AB]
#5 Occupational cervicobrachial disorders [TI/AB]	#15 therapeutic options [TI/AB]
#6 Occupational Back Pain [TI/AB]	#16 medication [TI/AB]
#7 Occupational health issues [TI/AB]	#17 medication side effects [TI/AB]
#8 Occupational diseases [TI/AB]	#18 OR/10-17
#9 OR/1-8	#19 9 AND 18
#10 Prevention [TI/AB]	

## References

[1] Wai EK, Roffey DM, Bishop P, Kwon BK, Dagenais S (2010). Causal assessment of occupational bending or twisting and low back pain: results of a systematic review. Spine J.

[2] (2023). Low back pain. https://www.who.int/news-room/fact-sheets/detail/low-back-pain.

[3] Luckhaupt SE, Dahlhamer JM, Gonzales GT, Lu ML, Groenewold M, Sweeney MH, Ward BW (2019). Prevalence, recognition of work-relatedness, and effect on work of low back pain among U.S. workers. Ann Intern Med.

[4] Ferguson SA, Merryweather A, Thiese MS, Hegmann KT, Lu ML, Kapellusch JM, Marras WS (2019). Prevalence of low back pain, seeking medical care, and lost time due to low back pain among manual material handling workers in the United States. BMC Musculoskelet Disord.

[5] Coenen P, Gouttebarge V, van der Burght AS, van Dieën JH, Frings-Dresen MH, van der Beek AJ, Burdorf A (2014). The effect of lifting during work on low back pain: a health impact assessment based on a meta-analysis. Occup Environ Med.

[6] Kim B, Yim J (2020). Core stability and hip exercises improve physical function and activity in patients with non-specific low back pain: a randomized controlled trial. Tohoku J Exp Med.

[7] Serranheira F, Sousa-Uva M, Heranz F, Kovacs F, Sousa-Uva A (2020). Low back pain (LBP), work and absenteeism. Work.

[8] Hartvigsen J, Hancock MJ, Kongsted A (2018). What low back pain is and why we need to pay attention. Lancet.

[9] Al-Otaibi ST (2015). Prevention of occupational back pain. J Family Community Med.

[10] Moher D, Shamseer L, Clarke M (2015). Preferred reporting items for systematic review and meta-analysis protocols (PRISMA-P) 2015 statement. Syst Rev.

[11] Ouzzani M, Hammady H, Fedorowicz Z, Elmagarmid A (2016). Rayyan—a web and mobile app for systematic reviews. Syst Rev.

[12] Ronzi Y, Roche-Leboucher G, Bègue C (2017). Efficiency of three treatment strategies on occupational and quality of life impairments for chronic low back pain patients: is the multidisciplinary approach the key feature to success?. Clin Rehabil.

[13] Movahedi M, Ghafari S, Nazari F, Valiani M (2017). The effects of acupressure on pain severity in female nurses with chronic low back pain. Iran J Nurs Midwifery Res.

[14] Sharafkhani N, Khorsandi M, Shamsi M, Ranjbaran M (2016). The effect of an educational intervention program on the adoption of low back pain preventive behaviors in nurses: an application of the health belief model. Global Spine J.

[15] Borges TP, Kurebayashi LF, Silva MJ (2014). Occupational low back pain in nursing workers: massage versus pain. Rev Esc Enferm USP.

[16] Saper RB, Boah AR, Keosaian J, Cerrada C, Weinberg J, Sherman KJ (2013). Comparing once-versus twice-weekly yoga classes for chronic low back pain in predominantly low income minorities: a randomized dosing trial. Evid Based Complement Alternat Med.

[17] del Pozo-Cruz B, Parraca JA, del Pozo-Cruz J, Adsuar JC, Hill J, Gusi N (2012). An occupational, internet-based intervention to prevent chronicity in subacute lower back pain: a randomised controlled trial. J Rehabil Med.

[18] Jaromi M, Nemeth A, Kranicz J, Laczko T, Betlehem J (2012). Treatment and ergonomics training of work-related lower back pain and body posture problems for nurses. J Clin Nurs.

[19] Rantonen J, Karppinen J, Vehtari A (2018). Effectiveness of three interventions for secondary prevention of low back pain in the occupational health setting - a randomised controlled trial with a natural course control. BMC Public Health.

[20] Miki K, Ikemoto T, Hayashi K, Arai YC, Sekiguchi M, Shi K, Ushida T (2018). Randomized open-label [corrected] non-inferiority trial of acetaminophen or loxoprofen for patients with acute low back pain. J Orthop Sci.

[21] Liu Q, Liu X, Lin H, Sun Y, Geng L, Lyu Y, Wang M (2023). Occupational low back pain prevention capacity of nurses in China: a multicenter cross-sectional study. Front Public Health.

[22] Zhang C, Yang Z, Zhang H (2022). Psychometric evaluation of the Chinese version of occupational LowBack pain prevention behaviors questionnaire among clinical nurses: a validation study. Front Public Health.

[23] Iwakiri K, Takahashi M, Sotoyama M, Liu X, Koda S (2019). Priority approaches of occupational safety and health activities for preventing low back pain among caregivers. J Occup Health.

[24] Mohseni Bandpei MA, Ehsani F, Behtash H, Ghanipour M (2014). Occupational low back pain in primary and high school teachers: prevalence and associated factors. J Manipulative Physiol Ther.

[25] Alghadir AH, Al-Abbad H, Buragadda S, Iqbal A (2021). Influence of work-related safety and health guidelines on knowledge and prevalence of occupational back pain among rehabilitation nurses in Saudi Arabia: a 6-month follow-up study. Int J Environ Res Public Health.

[26] Lee SS, Choi Y, Pransky GS (2016). Extent and impact of opioid prescribing for acute occupational low back pain in the emergency department. J Emerg Med.

[27] Babadi ME, Nazari F, Safari R, Abdoli S (2016). The effect of reflexology on pain perception aspects in nurses with chronic low back pain in Isfahan. Iran J Nurs Midwifery Res.

[28] Patel HD, Uppin RB, Naidu AR, Rao YR, Khandarkar S, Garg A (2019). Efficacy and safety of combination of NSAIDs and muscle relaxants in the management of acute low back pain. Pain Ther.

[29] Tetsunaga T, Tetsunaga T, Tanaka M, Ozaki T (2015). Efficacy of tramadol-acetaminophen tablets in low back pain patients with depression. J Orthop Sci.

[30] Fox LM, Murakami M, Danesh H, Manini AF (2018). Battlefield acupuncture to treat low back pain in the emergency department. Am J Emerg Med.

[31] Kobus AM, Smith DH, Morasco BJ, Johnson ES, Yang X, Petrik AF, Deyo RA (2012). Correlates of higher-dose opioid medication use for low back pain in primary care. J Pain.

[32] Kuijer PP, Verbeek JH, Visser B (2014). An evidence-based multidisciplinary practice guideline to reduce the workload due to lifting for preventing work-related low back pain. Ann Occup Environ Med.

[33] Sowah D, Boyko R, Antle D, Miller L, Zakhary M, Straube S (2018). Occupational interventions for the prevention of back pain: overview of systematic reviews. J Safety Res.

[34] Lim YZ, Chou L, Au RT (2019). People with low back pain want clear, consistent and personalised information on prognosis, treatment options and self-management strategies: a systematic review. J Physiother.

[35] van Middelkoop M, Rubinstein SM, Verhagen AP, Ostelo RW, Koes BW, van Tulder MW (2010). Exercise therapy for chronic nonspecific low-back pain. Best Pract Res Clin Rheumatol.

[36] Choi BK, Verbeek JH, Tam WW, Jiang JY (2010). Exercises for prevention of recurrences of low-back pain. Cochrane Database Syst Rev.

[37] Gordon R, Bloxham S (2016). A systematic review of the effects of exercise and physical activity on non-specific chronic low back pain. Healthcare (Basel).

[38] de Campos TF, Maher CG, Fuller JT, Steffens D, Attwell S, Hancock MJ (2021). Prevention strategies to reduce future impact of low back pain: a systematic review and meta-analysis. Br J Sports Med.

[39] Hayden JA, Ellis J, Ogilvie R, Malmivaara A, van Tulder MW (2021). Exercise therapy for chronic low back pain. Cochrane Database Syst Rev.

[40] Urits I, Burshtein A, Sharma M (2019). Low back pain, a comprehensive review: pathophysiology, diagnosis, and treatment. Curr Pain Headache Rep.

[41] Craige EA, Memon AR, Belavy DL, Vincent GE, Owen PJ (2023). Effects of non-pharmacological interventions on sleep in chronic low back pain: a systematic review and meta-analysis of randomised controlled trials. Sleep Med Rev.

[42] George SZ, Fritz JM, Silfies SP (2021). Interventions for the management of acute and chronic low back pain: revision 2021: clinical practice guidelines linked to the international classification of functioning, disability and health from the academy of orthopaedic physical therapy of the American Physical Therapy Association. J Orthop Sports Phys Ther.

[43] Karmali RN, Bush C, Raman SR, Campbell CI, Skinner AC, Roberts AW (2020). Long-term opioid therapy definitions and predictors: a systematic review. Pharmacoepidemiol Drug Saf.

[44] Main CJ, Foster N, Buchbinder R (2010). How important are back pain beliefs and expectations for satisfactory recovery from back pain?. Best Pract Res Clin Rheumatol.

[45] Dario AB, Moreti Cabral A, Almeida L (2017). Effectiveness of telehealth-based interventions in the management of non-specific low back pain: a systematic review with meta-analysis. Spine J.

[46] Henrotin YE, Cedraschi C, Duplan B, Bazin T, Duquesnoy B (2006). Information and low back pain management: a systematic review. Spine (Phila Pa 1976).

[47] Stephenson NL, Dalton JA (2003). Using reflexology for pain management: a review. J Holist Nurs.

[48] De Oliveira GS Jr, Castro-Alves LJ, McCarthy RJ (2015). Single-dose systemic acetaminophen to prevent postoperative pain: a meta-analysis of randomized controlled trials. Clin J Pain.

[49] Peck J, Urits I, Peoples S (2021). A comprehensive review of over the counter treatment for chronic low back pain. Pain Ther.

[50] Gianola S, Bargeri S, Del Castillo G (2022). Effectiveness of treatments for acute and subacute mechanical non-specific low back pain: a systematic review with network meta-analysis. Br J Sports Med.

